# Fast-HBR: Fast hash based duplicate read remover

**DOI:** 10.6026/97320630018036

**Published:** 2022-01-31

**Authors:** Sami Altayyar, Abdel Monim Artoli

**Affiliations:** 1 Department of Computer Science, College of Computer and Information Sciences, King Saud University, P.O. Box 51178, Riyadh 11543, Saudi Arabia

## Abstract

The Next-Generation Sequencing (NGS) platforms produce massive amounts of data to analyze various features in environmental samples. These data contain multiple duplicate reads which impact the analyzing process efficiency and accuracy. We describe Fast-HBR, a fast and memory-efficient duplicate reads removing tool without a reference genome using de-novo principles. It uses hash tables to represent reads in integer value to minimize memory usage for faster manipulation. Fast-HBR is faster and has less memory footprint when compared with the state of the art De-novo duplicate removing tools. Fast-HBR implemented in Python 3 is available at https://github.com/Sami-Altayyar/Fast-HBR.

## Background:

The number of the publicly available NGS projects tripled from 1200 in 2017 to 3500 in 2020 [1-2]. Therefore, preprocessing of data is essential to reduce the size of the data with an adequate level of data quality [3]. One of the preprocessing steps that reduce the dataset size is removing duplicate reads in the dataset. This step is essential for sequence-based algorithms since duplicate reads affect the algorithm accuracy [4]. Removing duplicate reads may reduce the assembly algorithms consumption of RAM [5]. Duplicate reads removal tools are either reference based or de novo. Some examples of de novo tools are CD-HIT [6], FastUniq [7] and Fulcrum [8]. Available de novo tools include NGS Reads Treatment [9], Nubeam-dedup [5], BioSeqZip [10] and Minirmd [11]. NGS Reads Treatment [9] is a hash-based tool that uses Cuckoo Filter [12] which is a probabilistic data structure. The authors elsewhere [5] developed the Nubeam-dedup tool that uses Nubeam [13] to represent each read as a number by calculating a product of matrices that represent nucleotides in the read. The BioSeqZip [10] tool starts by splitting the reads into small chunks, and then it sorts them alphabetically with memory limiting feature having long processing time. Minirmd [11] with the help of k-minimizer [14] clusters the reads into groups, where each group will contain reads that have the same k-minimizer in the same position. Therefore, it is of interest to describe Fast-HBR, a fast and memory-efficient duplicate reads removing tool without a reference genome using de-novo principles.

## Methodology:

Fast-HBR is implemented in Python 3. Therefore, it is platform-independent. The source code is available at https://github.com/Sami-Altayyar/Fast-HBR. It uses Python's built-in hash function to represent reads (in nucleotide or amino acid level) as an integer value. The reads hash value is stored in a set and each new read hash value will compare to the set items to decide if it is duplicate or not. The input files are either a single-end or paired-end, and it could process the files with reverse complement removing option or without it.

## Single-end files:

In single-end files, each read is independent; therefore its evaluation process will depend only on its hash value. Fast-HBR will starts by creating a set (UniqSet) to store all unique hash values. After that, it extracts from the input file one read at a time and then calculate the hash value (HV1) of the read. Depending on HV1 and the reverse complement removing option, Fast-HBR will have three cases. In the first case, if HV1 is in UniqSet, the read will consider a duplicate and will be discarded. In the second case, if HV1 is not in UniqSet and the reverse complement removing option is not activated, then HV1 will be added to UniqSet and the read will be written in the output file. In the third case, if HV1 is not in UniqSet and the reverse complement removing option is activated, Fast-HBR will calculate the hash value of the reverse complement of the read (HV2). If HV2 is in UniqSet the read will consider a duplicate and will be discarded. Otherwise, the read is unique and then only HV1 will be added to UniqSet and the read will be written in the output file.

**Figure 1:**
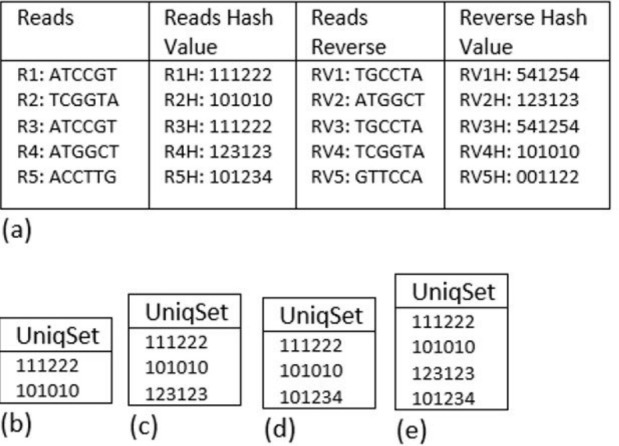
Fast-HBR methodology illustrated using an example.

We consider the input reads and their reverse and the hash values for the reads and the reverse as shown in Figure 1A. In the beginning, the reads R1 and R2 are unique and therefore their hash values would be added to UniqSet as in Figure 1B. For R3, its hash value (111222) is in UniqSet therefore R3 would be considered as duplicate read, and it will be discarded. Regarding read R4, the read hash value (123123) is not in UniqSet therefore if the reverse complement option is not active it will be considered a unique read and its hash value would be added to UniqSet as in Figure 1C, but if the reverse complement option is active, the hash value of the read reverse complement RV4 (101010) is in UniqSet and it will be considered as duplicate read and discarded.Finally, the read R5 hash value (101234) is not in UniqSet and its reverse complement hash value (001122) is not in UniqSet. Therefore, if the reverse complement option is active or not the read R5 is unique and the hash value of it (101234) would be added to UniqSet. Figure 1D shows the final UniqSet if the reverse complement option is active and Figure 1E if the reverse complement option is not active. Fast-HBR will not calculate the reverse complement hash unless it is necessary, which will minimize computational operations to the minimum. On the other hand, since we store only HV1 of unique reads in UniqSet, the number of elements in UniqSet will be less than or equal to the number of reads in the file. Consequently, the memory would be used efficiently, especially because the hash values in UniqSet are integers.

## Paired-end files:

For paired-end file processing, Fast-HBR would create a set (UniqSet) to store unique hash values. For each pair of reads i (R i1, R i2), if the reverse complement removing option is not activated, Fast-HBR would calculate the hash value (HV) as the hash of the concatenation of the two reads (Hash (R i1 concatenate R i2)). Then, if HV is present in UniqSet the reads pair (R i1, R i2) would be considered as a duplicate. Otherwise, the reads pair (R i1, R i2) is unique and will be written to the output file and HV would be added to UniqSet. The second case is when the reverse complement removing option is active as shown in Figure 1. Here, the change is the calculation of HV. It would be the sum of the hash value of R i1 plus the hash value of R i2. Therefore, if the pair reads in position (i) swapped in other position (j) in the file, they will have the same HV value and should be considered as a duplicate. Either with or without the reverse complement removing option, this methodology would guarantee that each pair of reads would represent by only one integer value. Because of that, the number of elements in UniqSet will be less than or equal to the number of pairs of reads, which lets Fast-HBR deal with memory more efficiently.

## Results and Discussion:

Results obtained using Fast-HBR is tabulated in Table 2(see PDF), Table 3(see PDF) and Table 4(see PDF). Comparisons with NGS Reads Treatment [9], Nubeam-dedup [5], BioSeqZip [10] and Minirmd [11] similar state of the art De novo tools are shown. The Linux bash command time was used to calculate the time spent by each tool and the tool's maximum memory usage. In this comparison, six datasets were used, three are single-end datasets (SRR10315305, SRR13555429 & SRR13555395) and three paired-end datasets (SRR681003, SRR837669, SRR6424061) and Table 1(see PDF) shows the datasets information. We run the tools on King Abdulaziz University's High Performance Computing Center (Aziz Supercomputer) (http://hpc.kau.edu.sa), where all tools run on normal nodes which equipped with 24 processors and 96GB memory. Because NGS Reads Treatment [9] and BioSeqZip [10] do not support the reverse complement removing option, we had to conduct two comparisons for each dataset. First, all five tools were compared without the reverse complement removing option, and the second comparison is only between Fast-HBR, Nubeam-dedup [5] and Minirmd [11] while with reverse complement removing option is activated.

NGS Reads Treatment [9] with a different number of threads (16, 24, 32) was very slow and was not able to complete the processing of five datasets (SRR13555429, SRR13555395, SRR681003, SRR837669, SRR6424061) because it exceeds the limited time for the job which is 48 hours. Minirmd [11] consumes a huge amount of memory and it failed to complete the processing of four datasets (SRR13555429, SRR13555395, SRR837669, SRR6424061) because of a memory error. Moreover, Nubeam-dedup [5] was able to process all datasets except SRR6424061 when the reverse complement removing option is activated because of memory error. On the other hand, Fast-HBR and BioSeqZip [10] were able to process all datasets successfully. We note that BioSeqZip [10] has the ability to limit the memory usage (default 4GB) and we try to increase its memory limit to 16GB, 32GB, and 64GB, but the tool failed to complete the process and cause a memory error, therefore, we run the tool with its default's memory limit.

Table 2(see PDF) shows the number of removed reads in each dataset after applying the tools. Minirmd [11] was the tool that removed the smallest number of duplicated reads. On the other hand, the remaining tools were able to remove the same number of duplicated reads except for Nubeam-dedup [5] in one dataset (SRR6424061) where it considered a slightly a greater number of reads as duplicated reads. The results of the tools regarding CPU time and memory footprint are tabulated in Table 3 and Table 4(see PDF). Table 3(see PDF) shows the results when the tools applied on the datasets without the reverse complement removing option, where Table 4(see PDF) contains the results when the reverse complement removing option is activated.

**Figure 2:**
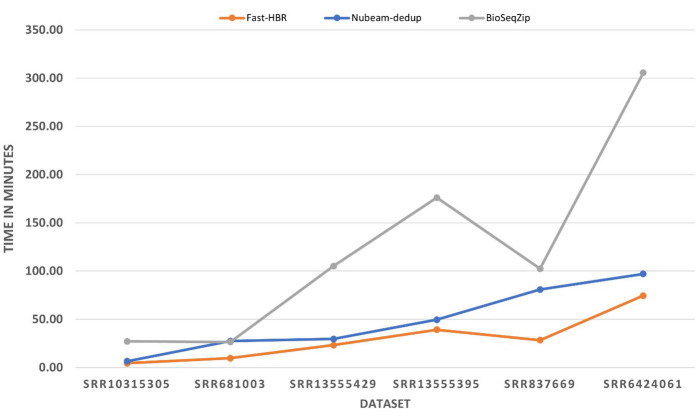
Processing time for the used datasets without reverse complement removing option.

**Figure 3:**
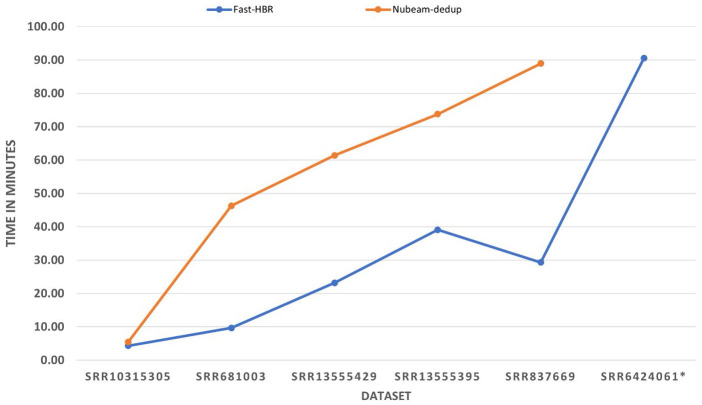
Processing time for the used datasets with reverse complement removing option. It should be noted that Nubeam-dedup was not able to complete processing SRR6424061 dataset.

Fast-HBR was the tool with the least CPU time in all single-end datasets in either case with or without reverse complement removing option. It was able to outperform the tool with the second least CPU time by a percentage that varies from 10% to 37%. In the paired-end datasets, Fast-HBR was the tool with the least CPU time in two of the three datasets and the outperform percentage in these two datasets varies from 23% to 67%. Generally, Fast-HBR was the tool with the least CPU time in ten out of twelve possible cases of processing datasets. Finally, the processing time for the tools when reverse complement is not activated is shown in Figure 2 while Figure 3 shows the processing time for the tools when reverse complement activated and here we should mention that NGS Reads Treatment [9] and Minirmd [11] are removed from the figures because they were not able to complete most of the datasets. BioSeqZip [10] consume almost the same memory amount in all datasets because of its memory limit control. Therefore, it has a smaller memory footprint than Fast-HBR in all datasets except SRR10315305. If we exclude BioSeqZip [10] because it caused memory error when we try to increase the memory limit, Fast-HBR consumes the least memory in all paired-end datasets with or without the reverse complement removing option. Moreover, when the reverse complement removing option is activated, Fast-HBR has the least memory footprint while processing all datasets. By comparing each tool's memory consumption when the reverse complement is not active (Table 3 - see PDF) and when the reverse complement is activated (Table 4 - see PDF), we noted that the amount of memory used by the Fast-HBR is almost unchanged whether the reverse complement option is enabled or not. On the other hand, when the reverse complement option is enabled the memory footprint of Nubeam-dedup [5] almost doubled.

## Conclusion:

We describe a de novo tool named Fast-HBR to remove duplicated reads in the meta-genomics data to reduce the dataset size which will benefit the meta-genomics analyzing pipelines. Fast-HBR represents each read to a single integer value by using hashing algorithms and hash tables for memory efficiency and speed. Fast-HBR shows the least computational requirement in validation. The CPU time required by it was less than the second-best tool Nubeam-dedup [5] by at least 10% and up to 67%. Moreover, Fast-HBR is the least memory consumption tool in all paired-end datasets using the reverse complement removing option.
